# The Correlation between Chemical Composition, as Determined by UPLC-TOF-MS, and Acute Toxicity of *Veratrum nigrum* L. and *Radix paeoniae alba*

**DOI:** 10.1155/2014/892797

**Published:** 2014-06-12

**Authors:** Xianxie Zhang, Yuguang Wang, Qiande Liang, Zengchun Ma, Chengrong Xiao, Hongling Tan, Yue Gao

**Affiliations:** Department of Pharmacology and Toxicology, Beijing Institute of Radiation Medicine, Taiping Road 27, Beijing 100850, China

## Abstract

The eighteen incompatible medicaments is an important theory in traditional Chinese medicine. The theory suggests that drugs in the eighteen incompatible medicaments can be toxic when used together. *Veratrum nigrum* L. and *Radix paeoniae alba* belong to the eighteen incompatible medicaments and have been prohibited for thousands of years. This study offers preliminary insight into the mechanism and chemical constituents responsible for the incompatibility and toxicity of these two agents. Specifically, we performed toxicology studies to identify and quantify the constituent substances of the two agents. Experiments revealed that acute toxicity increases when the dose of *V. nigrum* L. is higher than, or equal to, RPA. UPLC-TOF-MS analysis showed that, although the volumes of *V. nigrum* L. were the same, the content of some veratrum alkaloids changed significantly and had a trend toward a highly positive correlation (|*r*| ≥ 0.8) with toxicity. This suggests that the increased toxicity of the *V. nigrum* L. and RPA combination was due mainly to increased content of the special veratrum alkaloids. The cytotoxicity of veratridine in SH-SY5Y cells was decreased with increasing paeoniflorin concentrations. This study provides insight into the mechanism behind the incompatibility theory of TCM.

## 1. Introduction

Traditional Chinese medicine (TCM) is an important part of Chinese culture and makes significant contributions to the prosperity and health of the Chinese population. TCM has become more popular worldwide because of its efficacy and curative effects. However, it is important to ensure that these treatments are safe. Confirming the compatibility of TCM is currently the major method used to ensure its safety and efficacy [1]. TCM has formed a unique incompatibility theory over thousands of years, and the typical principles of prescriptions include the eighteen incompatible medicaments. This means that these specific agents can be toxic when used in combination [2]. However, some studies have used combinations of drugs in the eighteen incompatible medicaments to treat incurable diseases [2]. Therefore, it is important to determine whether these agents are incompatible when used in combination, and the reasons behind any incompatibility.

In this study, we used* Veratrum nigrum* L. (*V. nigrum* L.) and* Radix paeoniae alba* (RPA), which are two agents belonging to the eighteen incompatible medicaments whose concurrent use has been prohibited for thousands of years. Although there are no cases describing the compatibility of* V. nigrum* L. and RPA in modern Chinese medicine, fewer than 20 prescriptions using a combination of* V. nigrum* L. and RPA have been described to treat incurable diseases such as tumors, hemorrhoids, carbuncle, and breast carbuncle [3]. Therefore, it is necessary to determine whether the use of* V. nigrum* L. and RPA in combination should be prohibited, as well as the reasons for any incompatibility [4].

*V. nigrum* L. is the dried roots and rhizomes of* Veratrum nigrum* L. It has been used for medicinal purposes for thousands of years in China and was used in Europe during the Middle Ages [5] despite its well-known poisonous characteristics [4]. It is used to treat hypertension, stroke, excessive phlegm, and epilepsy. However, aqueous extracts are toxic and irritate the digestive tract mucosa, nucleus nervi vagi, and central nervous system [6, 7].

RPA, the dried root of* Paeonia lactiflora* Pall without bark, has been used as a medicinal herb in traditional Chinese medicine for centuries and exerts a wide range of pharmacological activities. In ancient pharmacology, RPA was used to calm liver wind, relieve pain, nourish blood, regulate menstrual functions, and suppress sweating [8]. In modern pharmacology, RPA decoctions could be used to treat rheumatoid arthritis, systemic lupus erythematosus, hepatitis, dysmenorrhea, muscle cramping and spasms, and long-standing fever [4, 9, 10].

In this study, UPLC/TOF MS, multivariate statistical analysis, and typical metabolomics approach were used to identify the key chemical markers that are responsible for the increased toxicity and the combination of* V. nigrum* L. and RPA. In addition, cellular and mouse acute toxicity assays were used to identify the toxic effects of this combination. The compatibility of* V. nigrum* L. and RPA has not yet been validated by modern science; therefore, it is important to provide preliminary insight into the mechanism of their reported incompatibility, as well as to identify and quantify their toxic chemical constituents.

## 2. Materials and Methods

### 2.1. Chemicals and Materials

RPA from Panan County (Zhejiang, China; lot 100714) and* V. nigrum* L. from Huajia County (Changchun, China; lot 100701) were purchased from Anhui BBCA Tongling Chinese herbal medicine company. Formic acid (CNW Technologies GmbH) and acetonitrile (Fisher Scientific, Fair Lawn, NJ, USA) were both of chromatographic purity. Deionized water was prepared using a Millipore water purification system. Veratridine (D00114760) was purchased from Merck (Merck KGaA, Germany), and paeoniflorin was purchased from Sigma-Aldrich. The MTS cell proliferation assay kit was purchased from Promega. Roswell Park Memorial Institute 1640 (RPMI-1640) and fetal bovine serum (FBS) were purchased from Gibco BRL (Invitrogen, USA).

### 2.2. Preparation of Decoctions

Based on a previous study, the LD_50_ of aqueous extracts of* V. nigrum* L. and RPA after intragastric administration were 2.566 g/kg and 160 g/kg, respectively [11]. In this study, the dose of* V. nigrum* L. was fixed at 2.566 g/kg, and the RPA dose varied from 0.2566 to 25.66 g/kg.

The doses of* V. nigrum* L. and RPA in each of 12 groups are shown in Table 1. Each group was extracted using deionized water (700 mL) for 1 h during microboiling under reflux. The extracts were filtered through three layers of gauze, and the drug extraction was then repeated. The filtrates were combined and were then concentrated to 100 mL at 60−70°C under reduced pressure. Samples were then shaken, calibrated, and stored at 4°C.

All decoctions were centrifuged at 13,000 rpm for 10 min using a Heraeus Labofuge 400R refrigerated centrifuge (Thermo Scientific, USA). The supernatants were then filtered through a 0.22 *μ*M aqueous microporous membrane and were stored at 4°C for UPLC-TOF-MS analysis. Groups were prepared in triplicate [12, 13].

### 2.3. Animals and Experimental Design

Seven-week-old Kunming (KM) mice (18−22 g) were obtained from the Experimental Animal Center of the Academy of Military Medical Sciences with the certificate of conformity SCXK-(Army) 2007-004. Animal subjects were housed at the SPF animal center of the Academy of Military Medical Sciences (Beijing, China) according to the regulations of the animal care committee. Mice were housed at a constant temperature of 25 ± 1°C with 50 ± 20% humidity, with a 12 h light/dark cycle and 10–15 air changes per hour. Mice were allowed free access to water and food during the experimental period.

Mice were acclimatized to the facilities and environment for 3 days before the experiments. Two hundred and forty mice were randomized into 12 groups (A–L), with 10 mice per group. Food was removed from all animals 12 h before the experiments. Mice received decoctions at 0.4 mL/10 g via gavage and were observed for 14 days.

### 2.4. UPLC-MS

#### 2.4.1. Liquid Chromatography

UPLC was performed on a Waters Acquity UPLC system (Waters, Milford, MA, USA) equipped with a binary solvent delivery system, an autosampler, and a photodiode-array detection (PDA) system. Chromatography was performed using a Waters ACQUITY BEHC_18_ column (100 mm × 2.1 mm, 1.7 *μ*m) [12] The mobile phase consisted of (A) water containing 0.1% formic acid and (B) acetonitrile containing 0.1% formic acid. The eluting conditions were as follows: isocratic 2% B (0-1 min), linear gradients of 2−5% B (1-2 min), 5–20% B (2–5 min), 20–30% B (5–7 min), 30–33% B (7–10 min), 33–36% B (10–13 min), 36–40% B (13–17 min), 40–100% B (17-18 min), 100–2% B (18-19 min), and 2% B (19-20 min). The flow rate was 0.5 mL/min. The sample chamber was maintained at 4°C, with the column at 45°C. The injection volume was 5 *μ*L [12, 14, 15].

#### 2.4.2. Mass Spectrometry

Mass spectrometry was performed using a Waters SYNAPT mass spectrometer equipped with an electrospray ionization (ESI) source. Samples were injected twice: once in positive ESI mode and once in negative ESI mode. The data acquisition range was 100–1500 Da. The lock spray reference scan frequency was 20 s, with a reference cone voltage of 30 V. The MS source temperature was set at 100°C, and the desolvation temperature was 450°C with a gas flow of 900 L/h. The lock mass compound was leucine enkephalin (200 pg/*μ*L), with an m/z of 556.2771 in the positive ion mode and 554.2615 in negative ion mode. The capillary voltages were set to 2.9 kV for ESI+ and 3 kV for ESI−. The cone voltage was 40 kV, and the collision energies were 6 V (trap) and 4 V (transfer), with 2.00 mL/min trap gas flow [16].

### 2.5. SH-SY5Y Cell Cultures and Cell Proliferation Assay

SH-SY5Y cells (ATCC, Manassas, USA) were cultured in RPMI-1640 supplemented with 10% FBS. Cells were maintained at 37°C in an incubator with a saturated humidity atmosphere of 95% air and 5% CO_2_. They were cultured in a 96-well plate and treated with different drug combinations. An MTS cell proliferation assay was performed, and the OD at 490 nm was read using a VICTOR X plate reader (Perkin Elmer, USA).

### 2.6. Data Analysis

The UPLC-TOF-MS data of all samples were analyzed using MassLynx4.1 software (Waters, Manchester, UK), and principal component analysis (PCA) was used for data analysis. Pearson correlation coefficients were used to identify relationships between the study parameters and mortality. The chemical markers in each group were identified using the* V. nigrum* L. and RPA chemical databases [12]. For data analyses, we compared the correlation between chemical composition and the acute toxicity of* V. nigrum* L. and RPA between the 12 groups. Experimental values are expressed as means ± standard deviations (SD). Statistical analyses were performed using two-tailed Student's *t*-tests. A value of *P* < 0.01 was considered statistically significant.

## 3. Results and Discussion

### 3.1. Mouse Acute Toxicity

Mice in the 12 groups were administered the decoctions by intragastric administration. Death occurred 5−10 min after administration. Toxicity was manifested predominantly as trembling, convulsions, and spasms. The major organs showed no obvious lesions at necropsy by the naked eye [6, 17, 18]. Mouse mortality is shown in Table 2.

**Figure 1:**
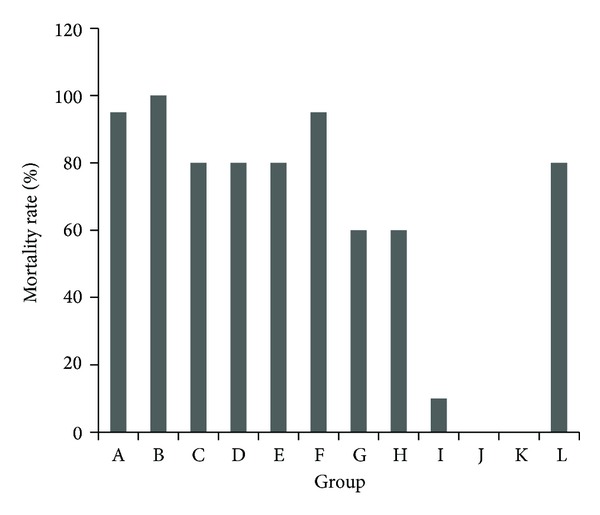
Total mortality of mice after the intragastric administration of decoctions containing different proportions of* V. nigrum* L. and RPA. The groups represent the combinations shown in Table 1.

Group L (with a* V. nigrum* L. dose of the LD_50_ 2.566 g/kg) had a mortality of 70%. Groups A−F were treated with the same dose of* V. nigrum* L., but the groups fed a lower ratio of RPA had a higher mortality than group L. Groups G−K had a higher proportion of RPA and lower mortality than group L, suggesting that toxicity was minimized. Groups B and F were the most toxic with mortalities of 100% and 95%, respectively. Overall analyses showed that mortality increased when the dose of* V. nigrum* L. was less than the dose of RPA. Conversely, toxicity decreased when the proportion of RPA increased. The trends in mortality are shown in Figure 1.

**Figure 2:**
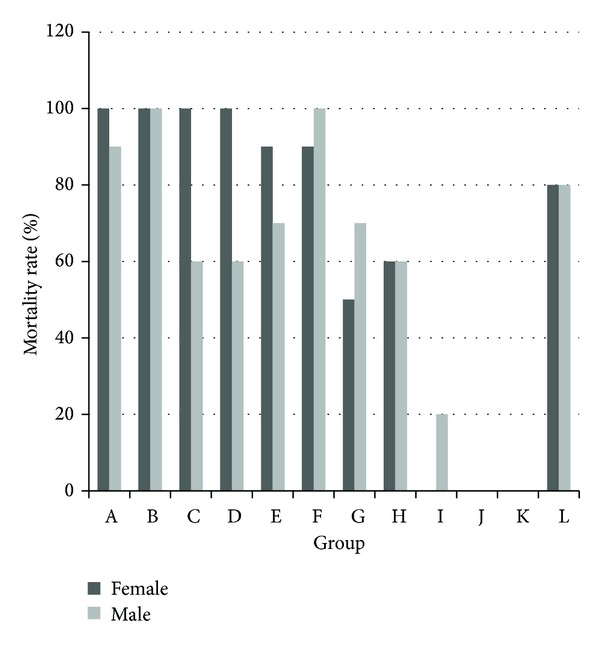
Mortality of male and female mice after the intragastric administration of decoctions containing different proportions of* V. nigrum* L. and RPA. The groups represent the combinations shown in Table 1.

Female mouse mortality decreased with increasing proportions of RPA, whereas males mortality exhibited two peaks at groups B and F, which both had 100% (Figure 2).

### 3.2. Identification and Quantification of the Chemical Components of* V. nigrum* L. and RPA

**Figure 3:**
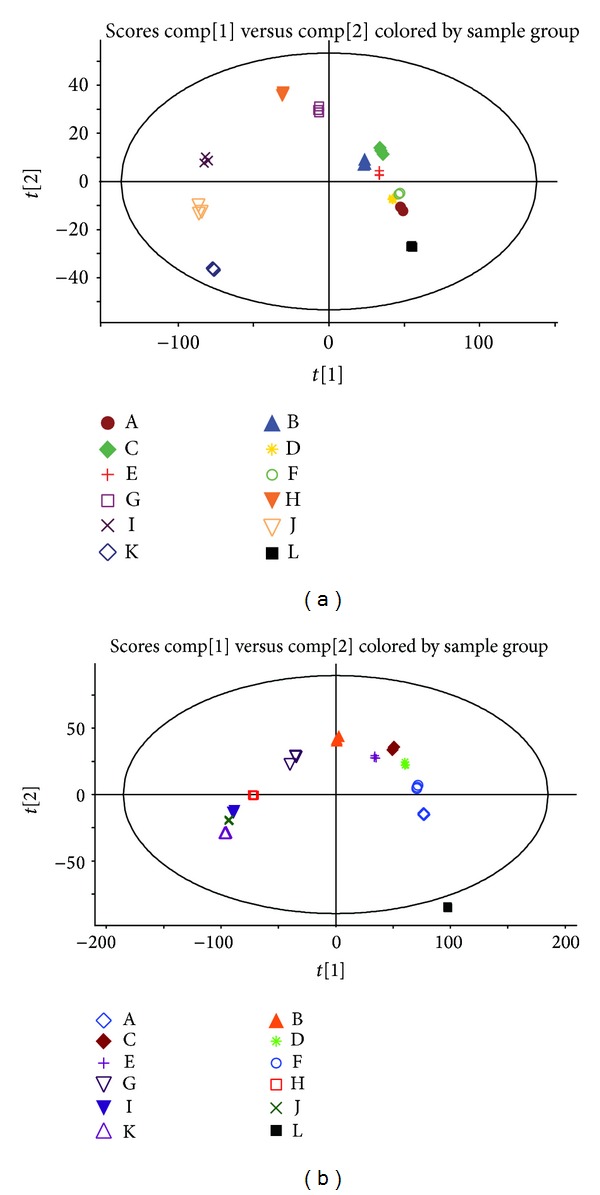
BBThe OPLS-DA score plot of 36 samples obtained using Pareto scaling with mean centering. (a) Positive ion mode. (b) Negative ion mode. The same score plots represent the same group. Both the positive and negative ion modes showed that the samples were clearly clustered into 12 groups. Replicates of the 12 groups could be distinguished easily.

To explain the cause of acute toxicity of the combined use of* V. nigrum* L. and RPA, we compared the chemical composition of the 12 groups using a supervised orthogonal partial least squared discriminant analysis (OPLS-DA). After Pareto scaling with mean centering, the data from both the positive and negative ion modes were displayed as scores plots (Figure 3). The scores plots clearly revealed that the samples were clustered into 12 groups; replicates of same group were comparable, but the 12 groups could be distinguished easily. This suggests that the changes in chemical compositions of the 12 groups were consistent, and the experiment was reproducible.

**Figure 4:**
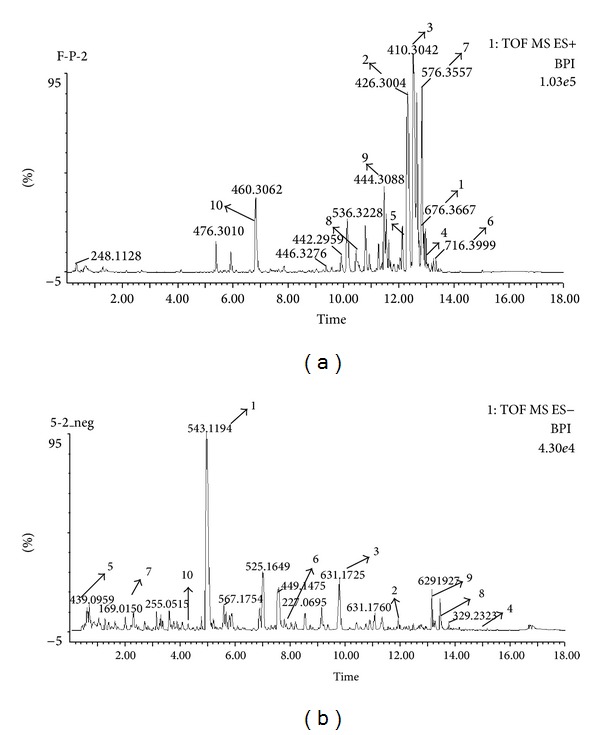
Representative BPI chromatograms. (a) Positive ion mode. (b) Negative ion mode. Some of identified constituents (1–10) are labeled in the BPI chromatogram, and the numbers correspond to Tables 3 and 4.

UPLC-TOF-MS is a rapid, specific, and sensitive method used to identify and quantify individual components. Representative BPI chromatograms of the 36 samples in the positive and negative mode ESI are shown in Figure 4.

The contents of some special alkaloids change significantly in different proportion decoctions. To determine which constituents contributed to the differences in toxicity observed with the 12 decoctions, statistical analyses were performed using Pearson's correlation, and the correlation coefficient (*r*) described the degree of linear correlation between two variables: total mouse mortality and the chemical composition of the 12 decoctions. The *r* values were between −1 and +1; *r* > 0 indicates that two variables were positively correlated, whereas *r* < 0 indicates a negative correlation. The larger the absolute value, the higher the correlation; therefore we defined *r* = 0.90–1.00 as extremely related and *r* = 0.80–0.89 as highly related. We extracted the relevant chemical component data when *r* ≥ 0.8 and identified the individual components using the RPA and* V. nigrum* L. chemical composition databases.

According to Pearson's correlation coefficient, 131 compositions had an extremely positive relationship (*r* ≥ 0.9) with toxicity. However, only 14 of these could be identified and all came from* V. nigrum* L. In addition, 35 components were highly positively correlated (*r* ≥ 0.8) to acute toxicity, and these constituents contributed most of the toxicity identified in the 12 decoctions. Therefore, we hypothesized that these components might play important roles in the acute toxicity of this drug combination. We identified that these compounds were all veratrum alkaloids that were detected in each sample in various ionized forms. The chemical components with *r* ≥ 0.8 are shown in Table 3 [7, 19–24].

**Figure 5:**
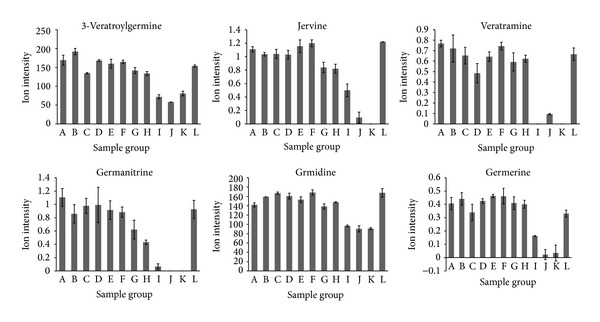
Ion intensity trend plots for 3-veratroylgermine, jervine, veratramine, germanitrine, germidine, and germerine, which were highly positively correlated (*r* ≥ 0.9393) with toxicity.

**Figure 6:**
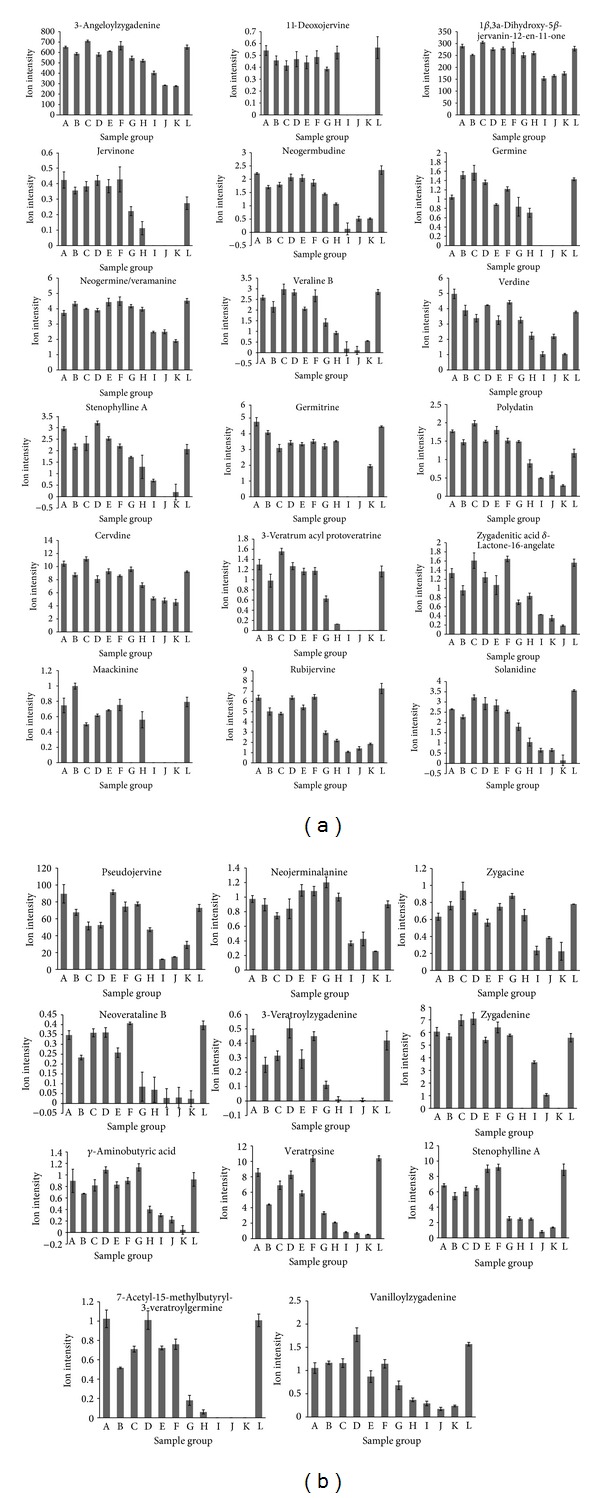
Ion intensity trend plots for the remaining 29 chemical components that were highly positively correlated (*r* ≥ 0.8) with toxicity.

3-Veratroylgermine, jervine, veratramine, germanitrine, germidine, and germerine were the main chemical components that were associated with acute toxicity, with Pearson's correlation coefficients of 0.9779, 0.9661, 0.9589, 0.9581, 0.9402, and 0.9393, respectively. These compounds are all veratrum alkaloids, and the changes in their content are shown in Figure 5. The other 29 components that were highly positively correlated (*r* ≥ 0.8) with acute toxicity are shown in Figure 6.

Interestingly, Pearson's correlation coefficient identified 24 RPA components that were extremely negatively correlated (*r* ≤ −0.9) with toxicity. The chemical components with *r* ≤ −0.9 are shown in Table 4 [25–33].

**Figure 7:**
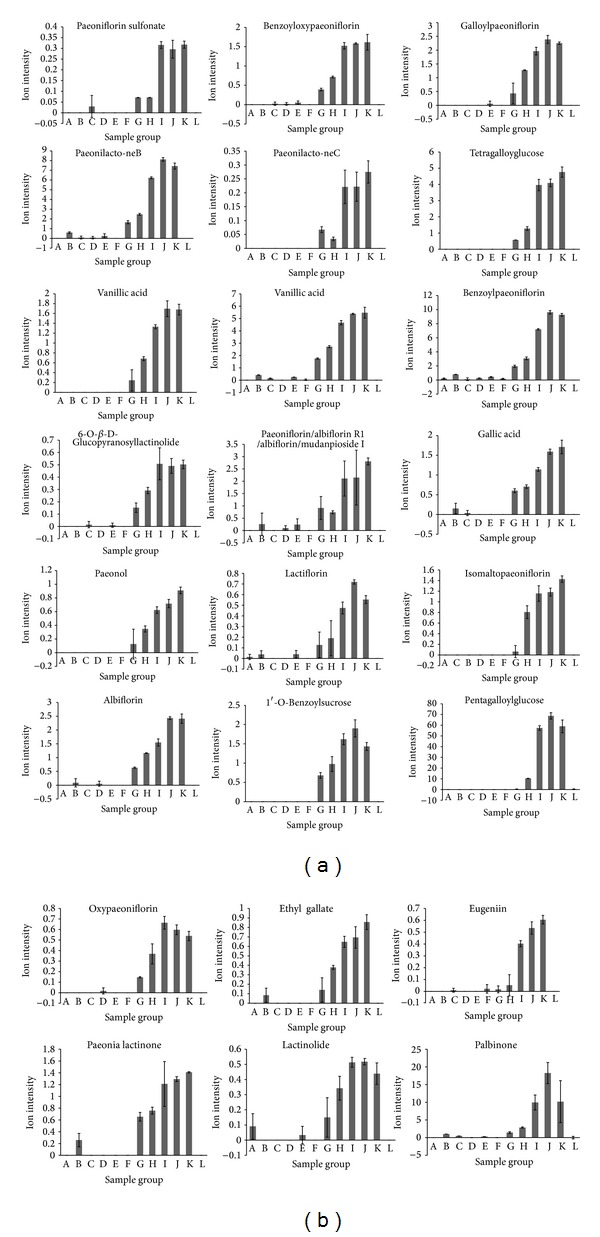
Ion intensity trend plots for the chemical components that were highly negatively correlated with toxicity (*r* ≥ 0.9).

The concentrations of these components increased with increasing amounts of RPA. Paeoniflorin sulfonate, benzoyloxy paeoniflorin, galloyl paeoniflorin, paeonilacto-neB, paeonilacto-neC, and tetragalloyglucose were the major RPA components that were identified, with Pearson's correlation coefficients of −0.9811, −0.9799, −0.9793, −0.9737, −0.9736, and −0.9734, respectively. The changes in content of the 24 components that were highly negatively correlated (*r* ≤ −0.9) with acute toxicity are shown in Figure 7.

### 3.3. Cell Proliferation Assay

**Figure 8:**
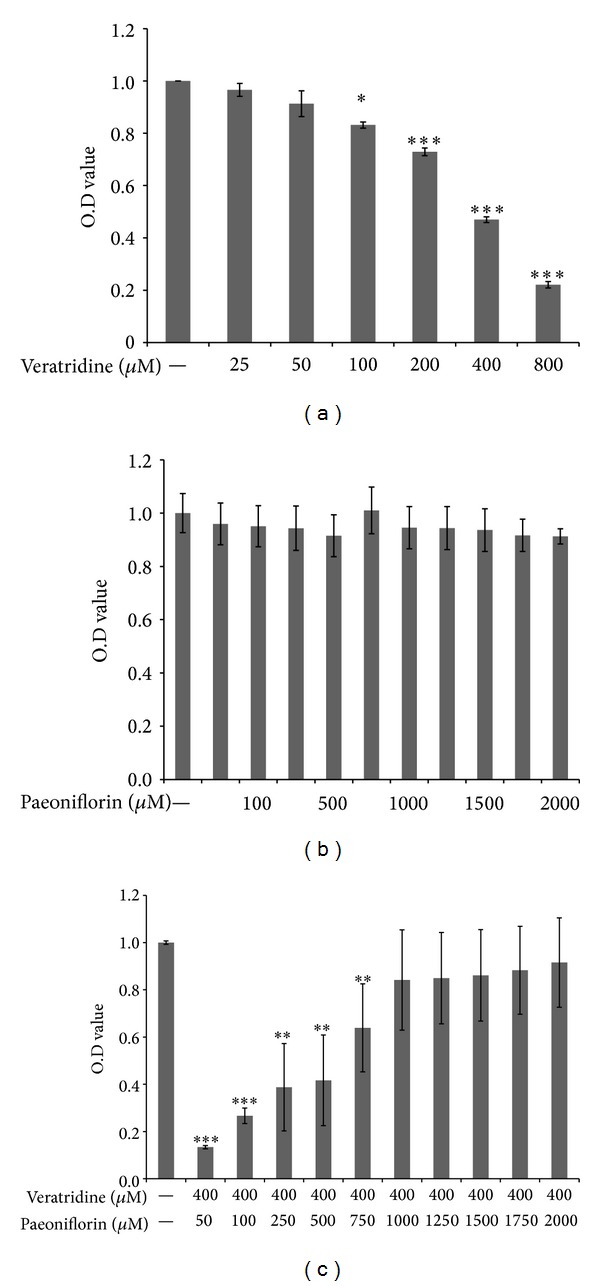
SH-SY5Y cell viability: SH-SY5Y cells were cultured with (a) veratridine (25–800 *μ*M), (b) paeoniflorin (50−2000 *μ*M), and (c) 400 *μ*M veratridine in combination with 50−2000 *μ*M paeoniflorin for 24 h. Cell viability was then assessed using MTS assays. Data are presented as mean ± S.D. Means of three independent experiments, Asterisks indicate a statistically significant difference compared with untreated cells. *P < 0.05; **P < 0.01; ***P < 0.001 versus controL.

To further validate the toxicity of the combination of* V. nigrum* L. and RPA on neurons, we used veratridine and paeoniflorin, the major pharmacologically active components of* V. nigrum* L. and peony for* in vitro* experiments. The effect of these two chemical components on the viability of SH-SY5Y cells was tested using MTS assays. As shown in Figures 8(a) and 8(b), veratridine had an LD_50_ of 450, whereas paeoniflorin exerted no significant toxicity at doses of 0–2000 *μ*M. Therefore, the dose of veratridine was fixed at 400 *μ*M, and the RPA dose was varied from 50 to 2000 *μ*M. SH-SY5Y cell cytotoxicity was increased when the concentration of paeoniflorin was decreased in the combination (Figure 8(c)). Therefore, paeoniflorin could offset the cytotoxicity caused by veratridine in SH-SY5Y cells [25].

## 4. Discussion

The eighteen incompatible medicaments (Shi Ba Fan) is a well-known traditional Chinese medicine (TCM) theory. It basically listed eighteen pairs of TCM herbal medicine that are not to be used in combination as they may cause fatal consequences. This principal has been used as guidance for general TCM practices for hundreds of years with little chemical comprehension. In this work, for the first time, we used the UPLC-TOF MS coupled with multivariate statistical analysis in an effort to gain scientific understanding for the causes of the incompatibility.

We previously reported that the LD_50_ of aqueous extracts of* V. nigrum* L. and RPA were 2.566 g/kg and 160 g/kg, respectively, after intragastric administration [11].* V. nigrum* L. extract was severely toxic, whereas RPA had minimal toxicity. Although the amount of* V. nigrum* L. in each group was unchanged, the mortality changed significantly with altered ratios of* V. nigrum* L. to RPA. When the doses of* V. nigrum* L. were equal to or higher than RPA, particularly ratios of 8 : 1 and 1 : 1, mouse mortality increased significantly. Therefore, components of* V. nigrum* L. were the major causative factors of toxicity. In addition, RPA modulated the toxicity of* V. nigrum* L. Therefore, clinical applications should be monitored carefully.

Mice died 5−10 min after drug administration. Toxicity manifested predominantly as trembling, convulsions, and spasms, but the major organs showed no obvious lesions at necropsy by the naked eye. Based on the toxic reactions and the time of death in the current study and according to the guidelines for Chinese natural medicine acute toxicity testing, we speculate that the toxicity was caused predominantly by suppression of the central nervous system and that* V. nigrum* L. was the major cause of death.* V. nigrum* L. water extracts exert toxic effects on the digestive tract mucosa, nucleus nervi vagi, and central nervous system [6, 17, 18]. Pharmacological studies of veratrum alkaloids have been performed previously and reported that these alkaloids stimulate the central nervous system and inhibit the brain. This results in spasms, convulsions, coma, drowsiness, and other symptoms of consciousness in animals, which is consistent with the symptoms of the mice in the current study [6, 17].

Veratrum alkaloid is the toxic component of* V. nigrum* L. We previously investigated the differently expressed genes in SH-SY5Y cells treated with veratridine using cDNA microarrays [34]. Data revealed that veratridine treatment altered the expression of MMP, caused Ca^2+^ concentration overload, increased reactive oxygen species production, enhanced LDH release, damaged the cell membrane, reduced SH-SY5Y cell viability, and induced apoptosis. Under oxidizing conditions, the MAPK signaling pathway is activated, which increases apoptosis. This study illustrates the neurotoxicity of the combination of* V. nigrum* L. and reveals the molecular mechanism behind the neurotoxic effects of* V. nigrum* L. (data not shown).

The amount of* V. nigrum* L. in each group was the same; the dose of RPA is in ascending order, and the chemical components of RPA are also increased, but the mortality of each group changed significantly with the increase of RPA; so we conclude that higher amount of RPA can have protection effect, which can offset the toxicity of combination of* V. nigrum* L. and RPA. And pharmacological studies have demonstrated that RPA exerts a wide range of pharmacological activities, including analgesic, sedative, anticonvulsant, and antispasmodic effects on the central nervous system. RPA could also inhibit writhing reactions and antagonize pentylenetetrazol-induced convulsions. The most important active ingredient in RPA is paeoniflorin, which has significant neuroprotective effects [26–34].

In this study, mouse acute toxicity experiments revealed that the mortality of mice changed significantly when the dose of* V. nigrum* L. was higher than or equal to that of RPA, particularly at ratios of* V. nigrum* L.: RPA of 8 : 1 and 1 : 1. UPLC-Q-TOF/MS with automated data analysis used to identify and quantify the specific chemical components. According to Pearson's correlation coefficient, most of the chemical components that were positively correlated with toxicity were from* V. nigrum* L. We conclude that the major veratrum alkaloids of* V. nigrum* L. caused acute toxicity and death in mice. When the doses of* V. nigrum* L. were higher than or equal to RPA, the main chemical components of* V. nigrum* L. and the toxicity of the decoctions were increased. This suggests that* V. nigrum* L. and RPA have opposing roles. Additional experiments revealed that the cytotoxicity of veratridine in SH-SY5Y cells was decreased with increasing concentrations of paeoniflorin. Therefore, the clinical use of these agents should be considered carefully. This study also provides information to help improve the incompatibility theory of TCM and introduces novel ideas for further studies on the development and application of TCM.

When preparing* V. nigrum* L. and RPA decoctions, a lower amount of RPA contributes to the dissolution of veratrum alkaloids. Conversely, higher amounts of RPA can exert neuroprotective effects, which can offset the toxicity caused by the combination of* V. nigrum* L. and RPA. Under these conditions, dissolution is the most important factor for* V. nigrum* L. toxicity. The results of cell-based experiments also demonstrated that paeoniflorin could offset the neurotoxic effects of veratridine when treated in combination, which excluded the factor of dissolution in preparation.

## Figures and Tables

**Table 1:** Doses of *V. nigrum *L. and RPA in the 12 decoction groups.

Group	*V. nigrum *L. dose (g)	RPA dose (g)	Water (mL)	Final volume (mL)	Drug concentration ratio (*V. nigrum *L.: RPA)
A	6.415	0.6415	700	100	10 : 1
B	6.415	0.802	700	100	8 : 1
C	6.415	1.069	700	100	6 : 1
D	6.415	1.604	700	100	4 : 1
E	6.415	3.208	700	100	2 : 1
F	6.415	6.415	700	100	1 : 1
G	6.415	12.83	700	100	1 : 2
H	6.415	25.66	700	100	1 : 4
I	6.415	38.49	700	100	1 : 6
J	6.415	51.32	700	100	1 : 8
K	6.415	64.15	700	100	1 : 10
L	6.415	0	700	100	—

**Table 2:** Mice mortality after the intragastric administration of different proportion decoctions of *V. nigrum *L. and RPA.

Group	*V. nigrum *L. dose (g)	RPA dose (g)	Crude drug concentration ratio	Female mortality	Male mortality	Total mortality
A	6.415	0.6415	10 : 1	100%	90%	95%
B	6.415	0.802	8 : 1	100%	100%	100%
C	6.415	1.069	6 : 1	100%	60%	80%
D	6.415	1.604	4 : 1	100%	60%	80%
E	6.415	3.208	2 : 1	90%	70%	80%
F	6.415	6.415	1 : 1	90%	100%	95%
G	6.415	12.83	1 : 2	50%	70%	60%
H	6.415	25.66	1 : 4	60%	60%	60%
I	6.415	38.49	1 : 6	0	20%	10%
J	6.415	51.32	1 : 8	0	0	0
K	6.415	64.15	1 : 10	0	0	0
L	6.415	0	0	70%	70%	70%

**Table 3:** The chemical components that were highly positively correlated with toxicity (*r* ≥ 0.8).

Number	*r*	tR (min)	Assigned identity	Molecular formula	Mean measured mass (Da)	Theoretical exact mass (Da)	Mass accuracy (ppm)
1	0.9779	12.98	3-Veratroylgermine	C_36_H_53_NO_11_	676.3667	675.8061	0.1805
2	0.9661	12.39	Jervine	C_27_H_39_NO_3_	426.3004	425.6035	2.3624
3	0.9589	12.61	Veretramine	C_27_H_39_NO_2_	410.3042	409.6041	2.9751
4	0.9581	13.43	Germanitrine	C_39_H_59_NO_11_	718.4169	717.8859	2.5015
5	0.9402	12.20	Germidine	C_34_H_53_NO_10_	636.3412	635.7853	0.5755
6	0.9393	13.40	Germerine	C_37_H_59_NO_11_	716.3999	693.8645	0.3408
7	0.9390	12.91	3-Angeloylzygadenine	C_32_H_49_NO_8_	576.3557	575.7334	1.2708
8	0.9311	10.35	11-Deoxojervine	C_27_H_41_NO_2_	412.3189	411.6199	6.4392
9	0.9240	1.57	1*β*,3*α*-Dihydroxy-5*β*-jervanin-12-en-11-one	C_27_H_41_NO_4_	444.3088	443.6187	2.4040
10	0.9237	6.96	Jervinone	C_27_H_37_NO_3_	424.2860	423.5876	2.0140
11	0.9182	11.25	Neogermbudine	C_37_H_59_NO_12_	732.3971	709.8639	4.9169
12	0.9143	4.25	Germine	C_27_H_43_NO_8_	510.3059	509.6322	1.5549
13	0.9131	4.18	Neogermine, Veramanine	C_27_H_43_NO_5_	462.3213	461.6340	1.4522
14	0.9034	11.58	Veraline B	C_27_H_45_NO_3_	432.3465	431.6511	2.9646
15	0.8978	11.16	Verdine	C_27_H_41_NO_5_	460.3056	459.6181	1.5249
16	0.8903	11.63	Stenophylline A	C_36_H_51_NO_11_	674.3474	673.7902	9.8655
17	0.8870	10.35	Germitrine	C_39_H_61_NO_12_	736.4278	735.9011	0.8288
18	0.8805	9.14	Polydatin	C_20_H_22_O_8_	429.0937	390.3839	3.4849
19	0.8738	10.81	Cervdine	C_32_H_49_NO_9_	592.3498	591.7328	2.0608
20	0.8675	12.34	3-Veratrum acyl protoveratrine	C_36_H_51_NO_12_	690.3482	689.7896	1.0609
21	0.8671	12.89	Zygadenitic acid *δ*-lactone-16-angelate	C_32_H_47_NO_8_	574.3408	573.7175	4.8884
22	0.8652	12.32	Maackinine	C_39_H_59_NO_11_	718.4171	717.8859	0.6797
23	0.8641	12.38	Rubijervine; rubivirine; etioline; epirubijervine; Isorubijervine	C_27_H_43_NO_2_	414.3357	413.6358	3.6827
24	0.8639	10.49	Solanidine	C_27_H_43_NO_3_	430.3308	429.6352	3.0494
25	0.8492	10.57	Pseudojervine	C_32_H_49_NO_8_	588.3525	587.7441	1.9710
26	0.8444	11.81	Neojerminalanine	C_39_H_61_NO_13_	752.4244	751.9005	3.0014
27	0.8379	8.43	Zygacine	C_29_H_45_NO_8_	536.3223	535.6695	0.0000
28	0.8358	13.20	Neoverataline B	C_27_H_42_N_2_O_9_	556.3260	538.6304	4.079
29	0.8201	12.80	3-Veratroylzygadenine	C_36_H_51_NO_10_	696.3187	657.7908	5.3469
30	0.8176	5.83	Zygadenine	C_27_H_43_NO_7_	494.3110	493.6328	1.5434
31	0.8176	0.54	*γ*-Aminobutyric acid	C_4_H_9_NO_2_	246.0978	103.1198	1.6121
32	0.8160	12.73	Veratrosine	C_33_H_49_NO_7_	616.3528	571.7447	6.8329
33	0.8128	13.22	Stenophylline A	C_37_H_55_NO_10_	674.3901	673.8333	0.5430
34	0.8119	13.34	7-Acetyl-15-methylbutyryl-3- veratroylgermine	C_43_H_61_NO_13_	838.3791	799.9433	1.3104
35	0.8017	12.13	Vanilloylzygadenine	C_35_H_49_NO_10_	644.3444	643.7643	1.5156

**Table 4:** The chemical components that were highly negatively correlated with toxicity (*r* ≤ −0.9).

Number	*r*	tR (min)	Assigned identity	Molecular formula	Mean measured mass (Da)	Theoretical exact mass (Da)	Mass accuracy (ppm)
1	−0.9811	4.95	Paeoniflorinsulfonate	C_23_H_28_O_13_S	543.1194	544.5256	3.3714
2	−0.9799	12.20	Benzoyloxy paeoniflorin	C_30_H_32_O_13_	599.1785	600.1843	0.3046
3	−0.9793	9.77	Galloyl paeoniflorin	C_30_H_32_O_15_	631.1725	632.1741	0.3856
4	−0.9737	14.79	Paeonilacto-ne B	C_17_H_18_O_6_	317.1750	318.1103	3.9209
5	−0.9736	0.56	Paeonilacto-ne C	C_10_H_12_O_4_	195.0496	196.0736	5.3192
6	−0.9734	8.09	Tetragalloyglucose	C_34_H_28_O_22_	787.1008	788.5729	5.6607
7	−0.9728	2.30	Vanillic acid	C_7_H_6_O_5_	169.0161	170.1195	3.7471
8	−0.9716	13.91	Desbenzoyl paeoniflorin	C_16_H_24_O_10_	375.1868	376.1369	4.4505
9	−0.9708	13.52	Benzoyl paeoniflorin	C_30_H_32_O_12_	583.1848	584.5679	0.6258
10	−0.9684	4.15	6-O-*β*-D-Glucopyranosyllactinolide	C_16_H_26_O_9_	361.1537	362.3722	9.3435
11	−0.9670	10.37	Paeoniflorin; albiflorin R1; albiflorin; mudanpioside I	C_23_H_28_O_11_	519.1261	480.4618	1.5284
12	−0.9655	11.67	Gallic acid	C_9_H_10_O_2_	151.0750	150.1745	5.9590
13	−0.9640	4.93	Paeonol	C_9_H_10_O_3_	165.0545	166.1739	4.0676
14	−0.9625	11.67	Lactiflorin	C_23_H_26_O_10_	480.1872	462.4465	0.4449
15	−0.9609	6.67	Isomaltopaeoniflorin	C_29_H_38_O_16_	643.2221	642.6024	2.6569
16	−0.9589	8.52	Albiflorin	C_23_H_27_O_11_	957.3118	479.4539	9.3723
17	−0.9582	6.79	1′-O-Benzoylsucrose	C_19_H_26_O_12_	464.1757	446.4025	2.3668
18	−0.9567	10.57	Pentagalloylglucose	C_41_H_32_O_26_	939.1166	940.6772	6.6292
19	−0.9562	8.02	Oxypaeoniflorin	C_23_H_28_O_12_	514.1934	496.4612	1.8992
20	−0.9544	11.58	Ethyl gallate	C_9_H_10_O_5_	221.0433	198.1727	3.2445
21	−0.9478	10.11	Eugeniin	C_41_H_30_O_26_	937.1010	938.6613	6.7086
22	−0.9395	5.85	Paeonilactinone	C_9_H_14_O_2_	153.0904	154.2063	7.5750
23	−0.9352	1.13	Lactinolide	C_10_H_16_O_4_	201.1111	200.2316	7.8907
24	−0.9173	13.73	Palbinone	C_22_H_30_O_4_	357.2047	358.4712	5.2115
